# Mitogen‐activated protein kinase p38 target regenerating islet‐derived 3*γ* expression is upregulated in cardiac inflammatory response in the rat heart

**DOI:** 10.14814/phy2.12996

**Published:** 2016-10-24

**Authors:** Hanna Säkkinen, Jani Aro, Leena Kaikkonen, Pauli Ohukainen, Juha Näpänkangas, Heikki Tokola, Heikki Ruskoaho, Jaana Rysä

**Affiliations:** ^1^ Research Unit of Biomedicine, Pharmacology and ToxicologyUniversity of OuluOuluFinland; ^2^ Department of PathologyCancer Research and Translational Medicine Research UnitUniversity of Oulu and Oulu University HospitalOuluFinland; ^3^ Division of Pharmacology and PharmacotherapyUniversity of HelsinkiHelsinkiFinland; ^4^ School of PharmacyUniversity of Eastern FinlandKuopioFinland

**Keywords:** Cardiac hypertrophy, inflammatory response, myocardial infarction, pressure overload

## Abstract

Regenerating islet‐derived 3*γ* (Reg3*γ*) is a multifunctional protein, associated with various tissue injuries and inflammatory states. Since chronic inflammation is characteristics also for heart failure, the aim of this study was to characterize Reg3*γ* expression in cardiac inflammatory conditions. Reg3*γ* expression was studied in experimental rat models of myocardial infarction (MI) and pressure overload in vivo. For cell culture studies neonatal rat cardiac myocytes (NRCMs) were used. In addition, adenovirus‐mediated gene transfer of upstream mitogen‐activated protein kinase (MAPK) kinase 3b and p38*α* MAPK in vivo and in vitro was performed. Reg3*γ* mRNA (12.8‐fold, *P* < 0.01) and protein (5.8‐fold, *P* < 0.001) levels were upregulated during the postinfarction remodeling at day 1 after MI, and angiotensin II (Ang II) markedly increased Reg3*γ* mRNA levels from 6 h to 2 weeks. Immunohistochemistry revealed that the Ang II‐induced expression of Reg3*γ* was localized into the cardiac fibroblasts and myofibroblasts of the proliferating connective tissue in the heart. Stretching and treatments with endothelin‐1, lipopolysaccharide (LPS), and fibroblast growth factor‐1 increased Reg3*γ* mRNA levels in NRCMs. SB203580, a selective p38 MAPK inhibitor, markedly attenuated LPS and mechanical stretch‐induced upregulation of Reg3*γ* gene expression. Moreover, combined overexpression of MKK3bE and WT p38*α* increased Reg3*γ* gene expression in cultured cardiomyocytes in vitro and in the rat heart in vivo. Our study shows that cardiac stress activates Reg3*γ* expression and p38 MAPK is an upstream regulator of Reg3*γ* gene expression in heart. Altogether our data suggest Reg3*γ* is associated with cardiac inflammatory signaling.

## Introduction

Heart failure (HF) is a complex clinical syndrome, and one of the leading causes of morbidity and mortality worldwide (Braunwald 2013). Chronic HF is associated with a combination of neurohormonal disturbance, imbalance between the expression of extracellular matrix proteins, increased oxidative stress and myocyte injury (Braunwald 2013). In addition, HF is associated with an ongoing inflammatory response (Levine et al. 1990), that is activated due to the myocardial damage of the failing heart and contributes to the worsening and progression of HF (Hartupee and Mann 2013; Frangogiannis 2014). Cardiac injury triggered immune response leads to infiltration of inflammatory cells into the myocardium and the increase in circulating and myocardial levels of proinflammatory mediators (Hartupee and Mann 2013), including members of the tumor necrosis factor (TNF) and interleukin (IL) superfamilies, expressed by all nucleated cell types in the myocardium (Knuefermann et al. 2004; Hartupee and Mann 2013). The cytokines are able to activate the proliferation of cardiac fibroblasts and myocytes as well as the extracellular matrix (Nicoletti and Michel 1999). Therefore, the continued inflammatory state in the heart results in development of diffuse fibrosis and loss of cardiomyocytes and contributes to adverse cardiac remodeling eventually leading to the development of HF (Ying et al. 2009; Jiang and Liao 2010). However, the complex molecular mechanisms underlying immunomodulatory processes in HF are poorly understood.

Regenerating (Reg) protein family comprises small C‐type lectin‐like secretory proteins, first identified in acute pancreatitis (Parikh et al. 2012). Members of Reg family are multifunctional proteins implicated in inflammation, cell proliferation, apoptosis, and antibacterial effects (Parikh et al. 2012). It has been shown that Reg family members have anti‐inflammatory effect in acute inflammation in pancreas (Zhang et al. 2004) and Reg3*γ*‐deficient mice had increased mucosal inflammatory responses to the pathogens in the ileum (Loonen et al. 2014). However, very little is known about the Reg proteins in the heart. Enhanced Reg1 gene expression has been described in response to acute myocardial infarction (MI) and pressure overload generated by aortic constriction in rats (Kiji et al. 2005) and in rat model of autoimmune myocarditis (Watanabe et al. 2008). In the heart, the studies of Reg3*γ* are limited to gene expression profiling screens reporting enhanced expression of Reg3*γ* in hypertrophied (Rysä et al. 2005), ischemic (Liu et al. 2009), and p38 MAPK overexpressing rat hearts (Tenhunen et al. 2006a) and in rat cardiomyocytes with autoimmune myocarditis (Watanabe et al. 2008). Recently, Reg3*β* was identified as an essential regulator of macrophage trafficking to the damaged heart (Lörchner et al. 2015). This study showed that activation of the Janus family tyrosine kinases (JAK) 1‐signal transducers and activators of transcription (STAT) 3 pathway was required for Reg3*β* expression in cardiomyocytes. In other tissues than the heart, STAT3 activation has been shown to regulate human REGI*α* and REGI*β* in salivary duct epithelial and pancreatic *β* cells (Yamauchi et al. 2015; Fujimura et al. 2016). However, the mechanisms regulating expression of Reg proteins are still poorly defined.

This study was designed to follow up the gene expression profiling findings during cardiac remodeling (Rysä et al. 2005; Tenhunen et al. 2006a; Watanabe et al. 2008; Liu et al. 2009). We examined the mechanisms and signaling pathways involved in the induction of Reg3*γ* gene expression by using models of hemodynamic stress in vivo and cultured rat neonatal cardiac myocytes.

## Methods

### Animals

The experiments of cardiac overload were performed in 2‐month‐old Sprague–Dawley male rats (SD‐rats) weighting 250–300 g. Male 12‐, 16‐, and 20‐month‐old spontaneously hypertensive rats (SHR) weighting 360–490 g of the Okamoto‐Aoki strain and their age‐matched controls Wistar Kyoto rats (WKY) were used as a model of chronic pressure overload. All the animals were from the colony of the Center of Experimental Animals at the University of Oulu, Finland. Spontaneously hypertensive rat strain was originally obtained from Mollegaars Avslaboratorium, Skensved, Denmark. All rats were kept in individual plastic cages with free access to tap water and normal rat chow. A 12‐h light and 12‐h dark environmental light cycle was maintained. The experimental design was approved by the Animal Care and Use Committee of the University of Oulu. All the experiments conform to the “European Convention for the Protection of Vertebrate Animals used for Experimental and other Scientific Purposes” (Council of Europe No. 123, Strasbourg 1985).

### Angiotensin II‐mediated hypertension, cardiac gene transfer, and MI in vivo

Angiotensin II (Ang II, 33 *μ*g kg^−1^ h^−1^) (Sigma‐Aldrich, St. Louis, MO) with and without Ang II receptor type 1 (AT_1_) blocker losartan (a generous gift from Merck, Kenilworth, NJ) (400 *μ*g kg^−1^ h^−1^) and the vehicle (0.9% NaCl) was administered via subcutaneously implanted osmotic minipumps (Alzet Osmotic pumps; DURECT Corporation, Cupertino, CA) in conscious rats for 6, 12, and 72 h, and 2 weeks as described previously (Suo et al. 2002). p38 mitogen‐activated protein kinase (MAPK) kinase inhibitor SB203580 (Tocris Bioscience, Bristol, UK) (1 mg kg^−1^ day^−1^) was administered i.p. with or without Ang II for 6 h. Using this model of experimental hypertension, administration of Ang II increases mean arterial pressure within 3 h and it remains significantly elevated throughout the 2‐week period (Suo et al. 2002). Blood pressure was measured using telemetry devices (model TA11PA‐C40; Data Sciences International, St Paul, MN).

Experimental MI was produced via ligation of left anterior descending coronary artery (LAD), as previously described (Tenhunen et al. 2006b), causing a thinning of the infarcted area, left ventricular dilatation, and impaired systolic function (Tenhunen et al. 2006b). The vehicles underwent the same surgical procedure without the ligation of LAD.

Adenovirus‐mediated gene transfer of the recombinant replication‐deficient adenoviruses containing the coding regions of the constitutively active mitogen‐activated protein kinase kinase 3b (MKK3b, RAdMKK3bE) and wild‐type (WT) and p38*α* MAPK (RAdp38*α*) into the left ventricle of male SD‐rats was performed as a local intramyocardial injection with a dose of 8 × 10^8^ pfu in a 100‐*μ*L volume, as previously described (Tenhunen et al. 2006a). RAd*lacZ*, which contains the *Escherichia coli β*‐galactosidase (*LacZ*) gene, was used as a control virus.

At the end of the experiments transthoracic echocardiography was performed using a commercially available Acuson Ultrasound System (SequoiaTM 512) and a 15‐MHz linear transducer (15 L8) (Acuson, Mountain View, CA) as previously described (Tenhunen et al. 2006b). After echocardiography, the animals were killed, all cardiac tissue samples were blotted dry, weighed, immersed in liquid nitrogen, and stored at −70°C until assayed as previously described (Rysä et al. 2005).

### Cell culture

Primary cultures of neonatal rat cardiac myocytes (NRCM) were prepared from 2‐ to 4‐day‐old SD‐rats, as described previously (Pikkarainen et al. 2003). Briefly, after digestion of ventricular tissue with collagenase type II (Millipore, Billerica, MA) (2 mg mL^−1^), cell suspension was preplated for 30–45 min. The nonattached cells or myocyte‐enriched cells were plated at the density of 2 × 10^5^ cm^−2^ on cell culture plates (Falcon; BD Biosciences, Fanklin Lakes, NJ) or on flexible‐bottomed collagen I‐coated elastomere plates (Bioflex; Flexcell International, Hillborough, NC) and cultured overnight in Dulbeccos's Modified Eagle Medium: Nutrient Mixture F‐12 (DMEM/F12) (Sigma‐Aldrich) containing 10% of heat‐inactivated fetal bovine serum (FBS) (Invitrogen, Carlsbad, CA), and thereafter in complete serum‐free medium (CSFM, containing DMEM/F‐12, 2.5 mg mL^−1^ bovine serum albumin, 1 *μ*mol L^−1^ insulin, 2.56 mmol L^−1^ l‐glutamine, 32 nmol L^−1^ selenium, 2.8 mmol L^−1^ sodium pyruvate, 5.64 *μ*g mL ^−1^ transferrin, 1 nmol L^−1^ T3, and 100 IU mL ^−1^ penicillin‐streptomycin).

The cells were treated with endothelin‐1 (ET‐1, 100 nmol L^−1^) (Sigma‐Aldrich), fibroblast growth factor 1 (FGF‐1, 50 ng mL ^−1^ with heparin 1 IU mL ^−1^) (R&D Systems, Minneapolis, MN) or lipopolysaccharide (LPS, 1 *μ*g mL ^−1^) (Sigma‐Aldrich) for 4–24 h. All substances were added to culture medium on the third day of the culture.

The cells were exposed to cyclic mechanical stretch for 24 and 48 h or 24 h with and without extracellular signal‐regulated kinase (ERK) inhibitor PD98059 (10 *μ*mol L^−1^), p38 MAPK inhibitor SB203580 (10 *μ*mol L^−1^) (Tocris Bioscience) or c‐jun N‐terminal kinase (JNK) inhibitor SP600125 (10 *μ*mol L^−1^) (Sigma‐Aldrich) by applying a computer controlled (Flexercell Strain Unit FX‐3000; Flexercell Int. Corp., McKeesport, PA) vacuum suction under the flexible‐bottomed collagen I‐coated 6‐well cell culture plates, as previously described (Pikkarainen et al. 2003). The frequency of stretch was 0.5 Hz with pulsation of 10–25% elongation of cells (Pikkarainen et al. 2003). After experiments the cells were quickly frozen with nitrogen oxide and stored at −70°C.

For in vitro adenoviral transfection experiment, cells plated on 12‐well plates were infected with adenoviruses overexpressing p38*α* (2 MOI) and Mkk3bE (2 MOI) or *LacZ* (4 MOI) for 2 days as previously described (Koivisto et al. 2014). The transfection was performed 24 h after plating of cells. The media was replaced every 24 h. After experiments the cells were washed twice with PBS and quickly frozen at −70°C.

### Isolation and analysis of RNA

Total RNA from tissue samples was isolated by the guanidine thiocyanate‐CsCl method and from cultured myocytes with TRIzol Reagent according to the manufacturer's protocol (Invitrogen) using Phase Lock Gel system (Eppendorf, Hamburg, Germany) as previously described (Rysä et al. 2005). For Northern blot analyses 20 *μ*g samples of RNA from ventricular tissue were separated on agarose‐formaldehyde gel electroforesis and transferred to MAGNA nylon membrane (Osmonics, Minnetonka, MN). PCR‐amplified probes corresponding to rat Reg3*γ* and 18S were labeled with [*α* ^32^P]dCTP, and the membranes were hybridized and washed, as described previously (Rysä et al. 2005). The membranes were exposed to Phosphor screens (GE Healthcare Life Sciences, Piscataway, NJ). The signal was detected with Molecular Imager FX Pro Plus equipment and measured using Quantity One software (Bio‐Rad Laboratories, Hercules, CA). The signals of Reg3*γ* gene were normalized to 18S in each sample.

When appropriate, rat Reg3*γ* and 18S mRNA levels were measured by real‐time quantitative RT‐PCR (QRT‐PCR) using TaqMan chemistry on an ABI 7700 Sequence Detection System (Applied Biosystems, Foster City, CA), as described previously (Tenhunen et al. 2006a). The sequences of forward (F) and reverse (R) primers and for fluorogenic probes (P) for RNA detection were as follows: rat Reg3*γ* (F) 5′‐AACAGTGGCCAAAACGTGTG‐3′, (R) 5′‐CCATCCACCTCTGTTGGGTT‐3′, (P) 5′‐Fam‐CCCAGTGTTGGATCATGGAGCCCA‐Tamra‐3′ and rat 18S (F) 5′‐TGGTTG‐CAAAGCTGAAACTTAAAG‐3′, (R) 5′‐AGTCAAATTAAGCCGCAGGC‐3′, (P) 5′‐Fam‐CCTGGTGGTGCCCTTCCGTCA‐Tamra‐3′.

### Protein extraction

The tissue was broken in liquid nitrogen, and homogenized in a lysis buffer consisting of 20 mmol L^−1^ Tris (pH 7.5), 10 mmol L^−1^ NaCl, 0.1 mmol L^−1^ EDTA, 0.1 mmol L^−1^ EGTA, 1 mmol L^−1^ *β*‐glycerophosphate, 1 mmol L^−1^ Na3VO4, 2 mmol L^−1^ benzamidine, 1 mmol L^−1^ phenylmethanesulfonyl fluoride (PMSF), 50 mmol L^−1^ NaF, 1 mmol L^−1^ dithiothreitol (DTT), and 10 *μ*g mL^−1^ of each leupeptin, pepstatin, and aprotinin as previously described (Tenhunen et al. 2006b). The tissue homogenates were centrifuged at 800 × *g* in +4°C for 1 min. To separate the total protein fraction, 5× NEB was added to the tissue homogenate, following by centrifugation at 12,500 rpm for 20 min. The supernatant was stored in −70°C until assayed.

To extract total protein from cultured cardiomyocytes, the cells were scraped in 100–150 *μ*L of 1× NEB buffer containing protease‐inhibitor cocktail (1:100 volume), phosphatase‐inhibitor cocktail (1:100 volume), and 1 mmol L^−1^ DTT (1:1000 volume). Cells were vortexed for 20 sec and centrifuged 12,500 rpm for 20 min at +4°C. The supernatant was collected as the total protein fragment and stored in −70°C until assayed. Protein concentrations were determined by colorimetric assay (Bio‐Rad Laboratories).

### Western blotting

For Western blot 20–30 *μ*g of denaturized protein was loaded onto a Novex 4–20% Tris‐Glysine gradient gel (Invitrogen) and transferred to nitrocellulose filters (Schleicher & Schuell BioScience, Dassel, Germany). The membranes were blocked in Odyssey Blocking buffer – TBS (1:1) (LI‐COR Biosciences, Lincoln, NE) and incubated with anti‐Reg3*γ* polyclonal antibody of Reg3*γ* (1:500 in Odyssey Blocking Buffer – TBS) overnight at +4°C. Antibody against rat Reg3*γ* was produced in rabbits by GenScript Corporation (Piscataway, NJ). After 1‐h incubation with Alexa Fluor goat anti‐rabbit secondary antibody (Invitrogen) at a 1:5000 dilution, the protein amount was detected using Odyssey Infrared Detection (LI‐COR Biosciences). The bands were quantified with Quantity One software (Bio‐Rad Laboratories).

### Immunohistochemical analysis

For immunohistochemical analysis, the hearts were fixed in 10% buffered formalin solution. Transversal sections of the left ventricle were embedded in paraffin, and 5‐*μ*m‐thick sections were cut. Sections were deparaffinized in xylene and dehydrated in graded ethanol, and to identify tissue location of Reg3*γ* incubated with specific rabbit polyclonal anti‐Reg3*γ* at the dilution of 1:50, 1:100, and 1:500. The primary antibody was detected by a peroxidase‐conjugated Dako REAL^™^ EnVision^™^ Detection System, Peroxidase/DAB+ kit (Dako, Glostrup, Denmark) according to the manufacturer's instructions, and the samples were counterstained with hematoxylin.

### Statistical analysis

Results are expressed as mean ± standard error of mean (SEM). For the comparison between two groups, data were analyzed by Student's *t*‐test. One‐way analysis of variance (ANOVA) followed by the least significant difference post hoc test was used to determine the significance of differences in multiple comparisons. *P* < 0.05 was considered statistically significant.

## Results

### Reg3γ expression is upregulated in cardiac remodeling in vivo

**Figure 1:**
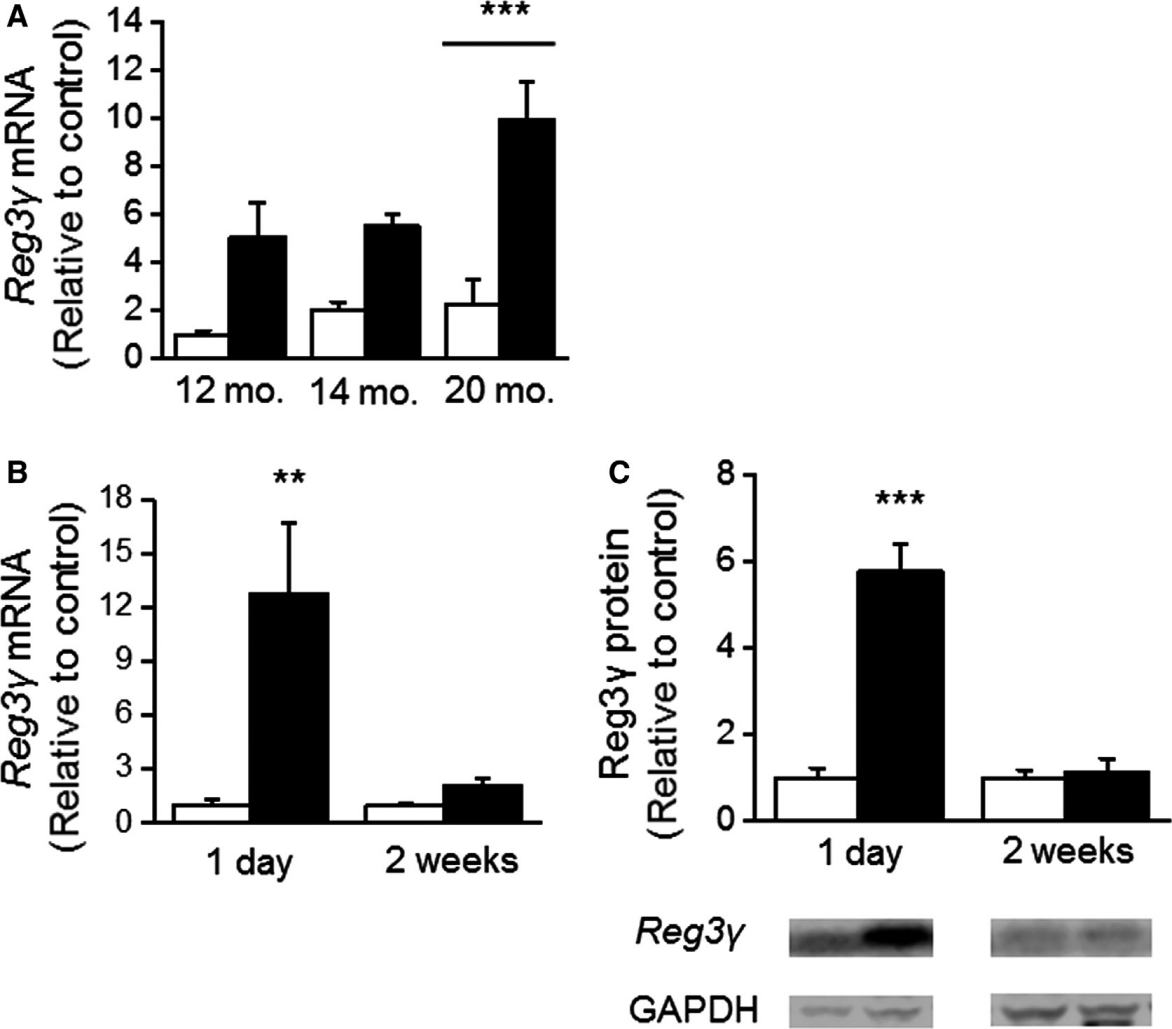
Cardiac regenerating islet‐derived 3*γ* (Reg3*γ*) gene expression in hypertrophied hearts and after myocardial infarction. Left ventricular Reg3*γ* gene expression in spontaneously hypertensive rats (SHR) and in their age‐matched controls Wistar Kyoto rats (WKY). (A) White columns, WKY; black columns, SHR. Gene expression results measured by Northern Blot analysis are expressed as a ratio of Reg3*γ* mRNA to 18S mRNA, mean ± SEM (*n* = 4–11). ***P < 0.001 20‐month‐old SHR versus age‐matched WKY rats (Student's *t*‐test). The effect of myocardial infarction (MI) on left ventricular Reg3*γ* mRNA (B) and protein (C) levels in rats after 1 day and 2 weeks after MI. Reg3*γ* was observed at 17 kDa, the values were normalized to GAPDH (36 kDa, total protein fraction), representative Western blots are shown. The controls went through the same invasive operation without the ligation of left anterior descending coronary artery. White columns, control; black columns, myocardial infarction. Gene expression results measured by Northern Blot analysis are expressed as a ratio of Reg3*γ* mRNA to 18S mRNA and total protein levels were assessed by Western blot analyses. Results are expressed as mean ± SEM (*n* = 5–8). ***P < 0.001, **P < 0.01, infarct versus control (Student's *t*‐test).

Comparable to the results obtained by the DNA microarray analysis of hypertrophied hearts of SHRs (Rysä et al. 2005), left ventricular Reg3*γ* mRNA levels were 4.4‐fold higher in 20‐month‐old SHR (*P* < 0.001) compared with age‐matched normotensive WKY rats (Fig. 1A). Next, to study the effect of cardiac postinfarction inflammatory response (Frangogiannis 2014) on cardiac Reg3*γ* expression, ligation of LAD was performed in normotensive rats (Tenhunen et al. 2006b). At 2 weeks intraventricular septum in diastole was significantly larger in infracted hearts than in sham‐operated hearts (1.3 ± 0.1 mm vs. 1.8 ± 0.1 mm; *P* < 0.001, *n* = 8). In addition, the significant decrease in fractional shortening (21 ± 2% vs. 42 ± 1%; *P* < 0.001) and ejection fraction (47 ± 3% vs. 77 ± 2%; *P* < 0.001, *n* = 8) was observed in infarcted versus sham‐operated hearts (*n* = 8 in both groups). Both Reg3*γ* mRNA and protein levels were markedly upregulated (*P* < 0.01 and *P* < 0.001, respectively) in the left ventricle at day 1 following MI returning to sham levels at 2 weeks after MI (Fig. 1B and C), indicating that postinfarction inflammatory response rapidly upregulates Reg3*γ* expression in the heart.

### Effect of Ang II‐induced hypertension on Reg3γ gene expression in the left ventricle

**Figure 2:**
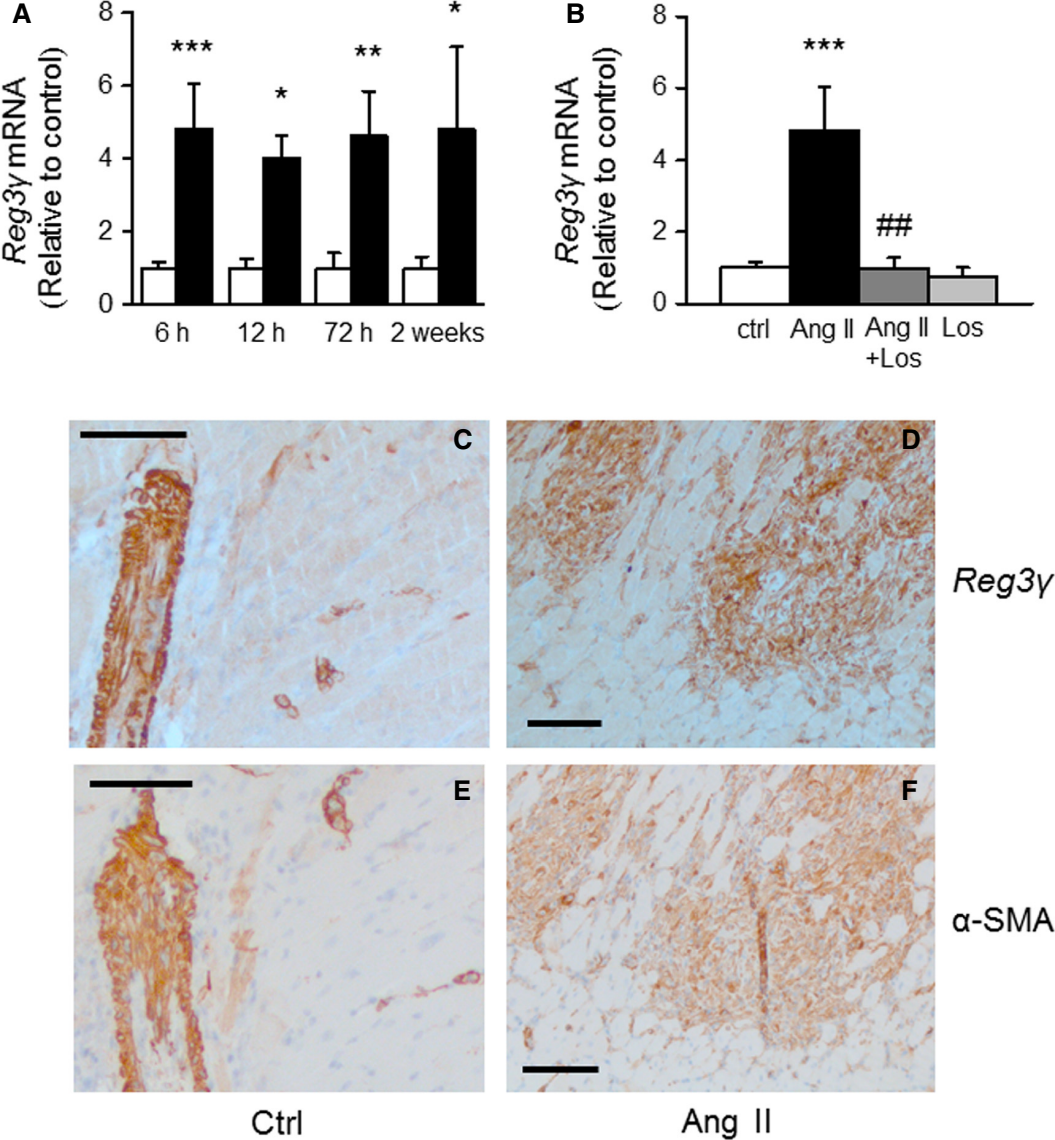
Angiotensin II (Ang II) regulates cardiac regenerating islet‐derived 3*γ* (Reg3*γ*) gene expression. The effect of subcutaneous administration of Ang II via osmotic minipumps for 6, 12, and 72 h, and 2 weeks on left ventricular Reg3*γ* mRNA levels (A). White columns, control; black columns, Ang II. (B) The effect of subcutaneous administration of Ang II, losartan (Los) and their combination for 6 h on left ventricular Reg3*γ* mRNA levels. White columns, control (ctrl); black columns, Ang II; dark gray column, Ang II + Los; light gray column, Los. Gene expression results measured by Northern Blot analysis are expressed as a ratio of Reg3*γ* mRNA to 18S mRNA, mean ± SEM (*n* = 6–9). *P < 0.05, **P < 0.01, ***P < 0.001, Ang II versus control (ctrl), ^##^ *P* < 0.01 Ang II + losartan versus Ang II (One‐way ANOVA followed by a least significant difference post hoc test). Representative images demonstrating cardiac Reg3*γ* (C, D) and alpha smooth muscle actin (*α*‐SMA) (E, F) expression in rats after 6 h‐infusion of Ang II. Control (ctrl) (C, E); Ang II (D, F). Magnification 100×, scale bar 100 *μ*mol L^−1^.

To characterize the changes in LV Reg3*γ* gene expression during pressure overload‐induced inflammation, we used a model of Ang II‐induced hypertension in conscious rats (Sciarretta et al. 2009). During Ang II infusion, mean arterial pressure was markedly elevated within 3 h (127 ± 11 mm Hg vs. 108 ± 11 mm Hg in Ang II vs. vehicle infused rats, *P* < 0.05, *n* = 10) and remained significantly increased throughout the 2‐week period (181 ± 5 mm Hg, *P* < 0.001 at the end of the experiment). Ang II infusion caused significant upregulation of Reg3*γ* gene expression already after 6 h (4.8‐fold, *P* < 0.001, *n* = 7) and remained significantly elevated throughout the 2 weeks’ follow‐up period (Fig. 2A). In addition, simultaneous infusion of the AT_1_‐receptor antagonist losartan completely abolished the upregulation of Reg3*γ* mRNA levels in the left ventricle (Fig. 2B).

To examine the cell types expressing Reg3*γ* in the heart, immunohistochemistry with Reg3*γ* antibody was performed in the adult rat heart. Immunohistochemistry revealed that the expression of Reg3*γ* localized into vascular smooth muscle cells (Fig. 2C) and the increased expression of Reg3*γ* at 6 h of Ang II infusion was localized into the fibroblast‐like spindle cells of the proliferating connective tissue (Fig. 2E). Moreover, the immunohistochemical staining pattern of Reg3*γ* (Fig. 2C and E) was identical to that of smooth muscle actin alpha (*α*‐SMA) (Fig. 2D and F) indicating that the spindle cells represented myofibroblasts.

### Regulation of Reg3γ gene expression in cultured neonatal cardiac myocytes

**Figure 3:**
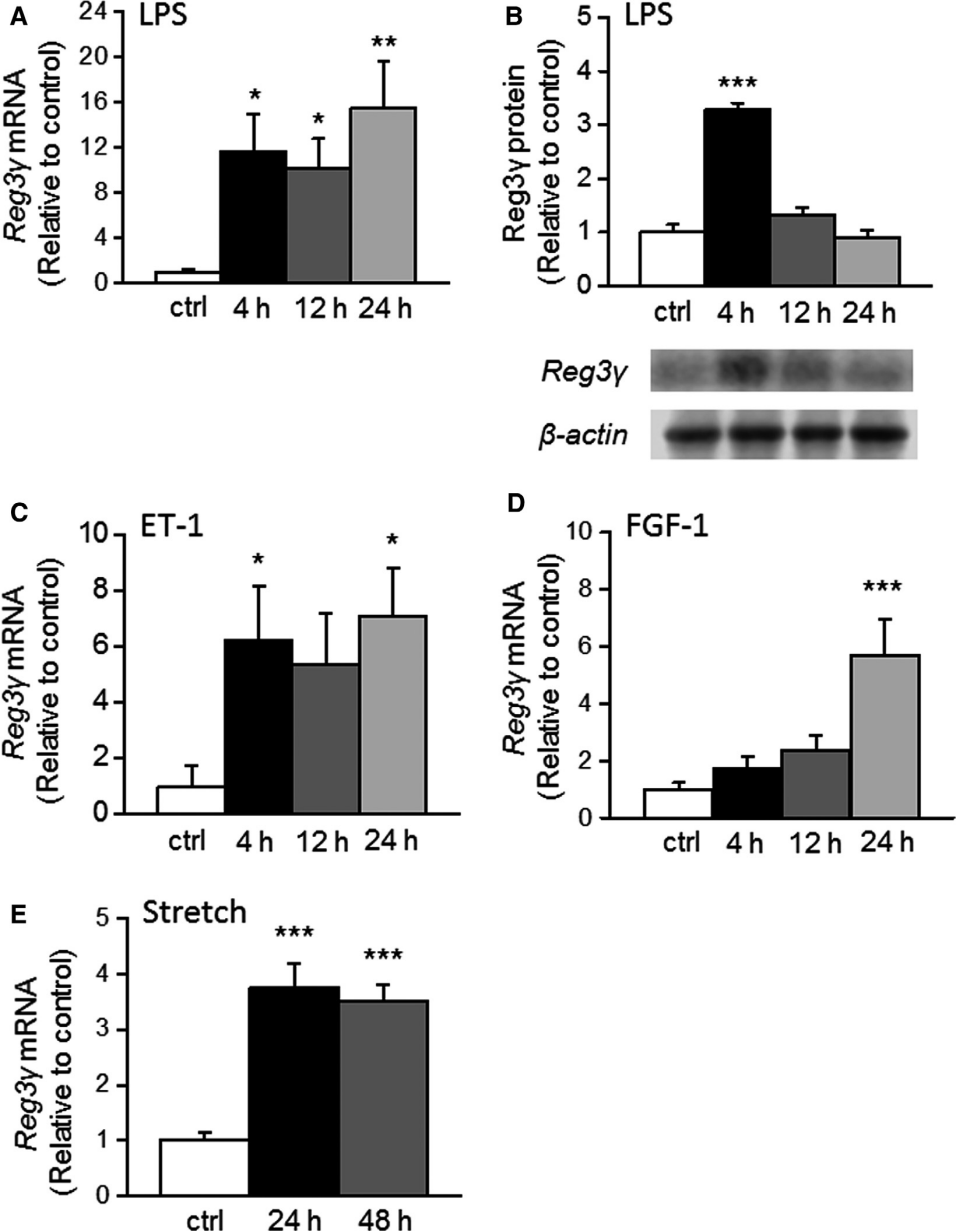
Regulation of regenerating islet‐derived 3*γ* (Reg3*γ*) expression in cultured neonatal rat ventricular myocytes. Ventricular myocytes were exposed to lipopolysaccharide (LPS) (A, B) endothelin‐1 (ET‐1) (C) and fibroblast growth factor ‐1 (FGF‐1) (D) for 4–24 h (*n* = 12–18). (E) Ventricular myocytes were exposed to cyclic mechanical stretch, in which the frequency of stretching was 0.5 Hz with pulsation of 10–25% elongation of cells for 24–48 h (*n* = 6). Reg3*γ* was observed at 17 kDa, the values were normalized to *β*‐actin (42 kDa, total protein fraction), and representative Western blots are shown. Gene expression results measured by quantitative RT‐PCR are expressed as a ratio of Reg3*γ* mRNA to 18S mRNA, mean ± SEM. *P < 0.05, **P < 0.01, ***P < 0.001 treated cells versus control (ctrl) (One‐way ANOVA followed by a least significant difference post hoc test).

To directly examine the regulation of Reg3*γ* expression in cardiomyocytes, we treated NRCMs with LPS, ET‐1, and FGF‐1 in vitro. LPS (1 *μ*g mL ^−1^) significantly increased Reg3*γ* gene expression from 4 h of LPS stimulation (11.6‐fold, *P* < 0.05) up to 24 h (Fig. 3A) and at protein levels at 4 h (3.3‐fold, *P* < 0.001) (Fig. 3B). Of note, in cardiomyocytes exposed to LPS Reg3*γ* protein levels peaked at 4 h, whereas mRNA levels were sustained. Although measuring mRNA is an indicator of gene regulation, the mRNA amount is not directly correlated with protein expression since posttranscriptional processes, action of miRNAs, and posttranslational management of proteins due to protein half‐life are involved in turning mRNA into protein. Most importantly, Reg3*γ* is a secreted protein (Parikh et al. 2012), so cellular protein levels do not necessarily reflect the total amount of protein. In addition, ET‐1 (100 nmol L^−1^) increased Reg3*γ* mRNA levels with a maximal increase (7.1‐fold, *P* < 0.05) at 24 h (Fig. 3C), and FGF‐1 (50 ng mL ^−1^) caused a 5.7‐fold increase in Reg3*γ* mRNA levels at 24 h (Fig. 3D). Furthermore, we used the well‐established in vitro model to study stretch‐activated changes Reg3 expression (Sadoshima et al. 1992), and noted a marked increase in Reg3*γ* mRNA levels was noted in response to 24‐ and 48‐h of cyclic mechanical stretching (*P* < 0.001) (Fig. 3E).

### The effect of p38 MAPK inhibition on Reg3γ gene expression

**Figure 4:**
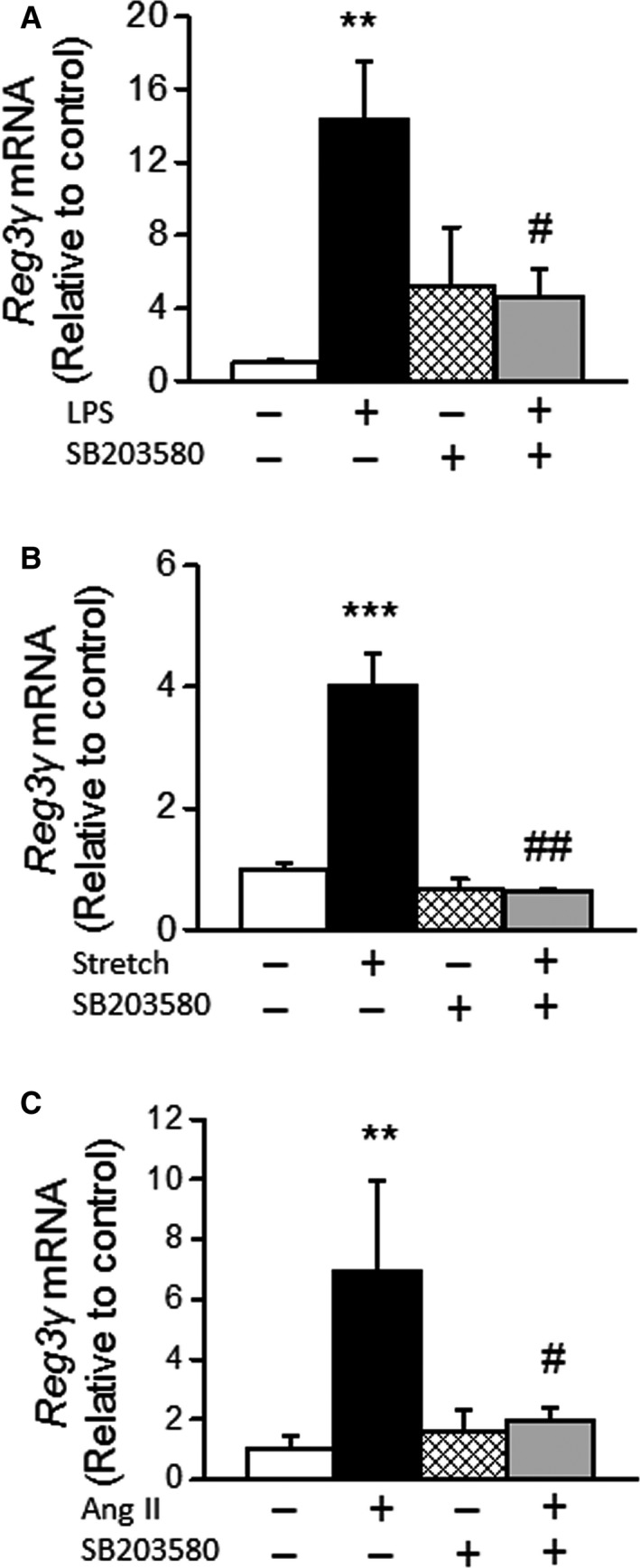
The effect of p38 MAPK inhibition on regenerating islet‐derived 3*γ* (Reg3*γ*) mRNA levels in cardiac myocytes and in the heart in vivo. (A) Neonatal cardiac myocytes were treated with lipopolysaccharide (LPS) with and without SB203580 for 4 h **P < 0.01, LPS versus control; ^##^ *P* < 0.01, LPS + SB203580 versus LPS (One‐way ANOVA followed by a least significant difference post hoc test), *n* = 18. (B) The effect of p38 MAPK inhibition on mechanical stretch‐induced Reg3*γ* gene expression in neonatal rat ventricular myocytes. Ventricular myocytes were exposed to cyclic mechanical stretch, in which the frequency of stretching was 0.5 Hz with pulsation of 10–25% elongation of cells for 24 h. ***P < 0.001, stretch versus control (one‐way ANOVA), *n* = 6. (C) Ang II was infused subcutaneously into rats with or without the p38 inhibitor SB203580 for 6 h. 0.9% NaCl was infused into control animals. White column, control; black column, Ang II; dark gray column, Ang II + SB203580; light gray column, SB203580. **P < 0.01, Ang II versus control; and #*P* < 0.05, Ang II + SB203580 versus Ang II (one‐way ANOVA), *n* = 4–7. All gene expression results measured by quantitative RT‐PCR are expressed as a ratio of Reg3 g mRNA to 18S mRNA, mean ± SEM.

Next, to investigate the possible signaling pathways mediating the activation of Reg3*γ* gene expression, we focused on p38 MAPK, since our earlier DNA microarray study identified Reg3*γ* as putative p38 MAPK target gene (Tenhunen et al. 2006a). First, the effect of pharmacological inhibition on LPS and mechanical stretch‐induced Reg3*γ* gene expression was studied in NRCMs. Inhibition of p38 MAPK with SB203580 (10 *μ*mol L^−1^) significantly reduced the both LPS (*P* < 0.01) and mechanical stretch (*P* < 0.05) stimulated Reg3*γ* gene expression in cultured cardiomyocytes (Fig. 4A and B). The inhibitors of ERK (PD98059) and JNK (SP600125) did not reduce the stretch‐induced upregulation of Reg3*γ* gene expression in cultured cardiomyocytes (data not shown). Finally, p38 MAPK inhibitor SB203580 was administrated simultaneously with Ang II‐ in SD‐rats. Administration of SB203580 reduced Ang II‐induced elevation of left ventricular Reg3*γ* gene expression by 72% (*P* < 0.05) at 6 h (Fig. 4C).

### The effect of adenovirus‐mediated p38 MAPK gene transfer on Reg3γ gene expression

**Figure 5:**
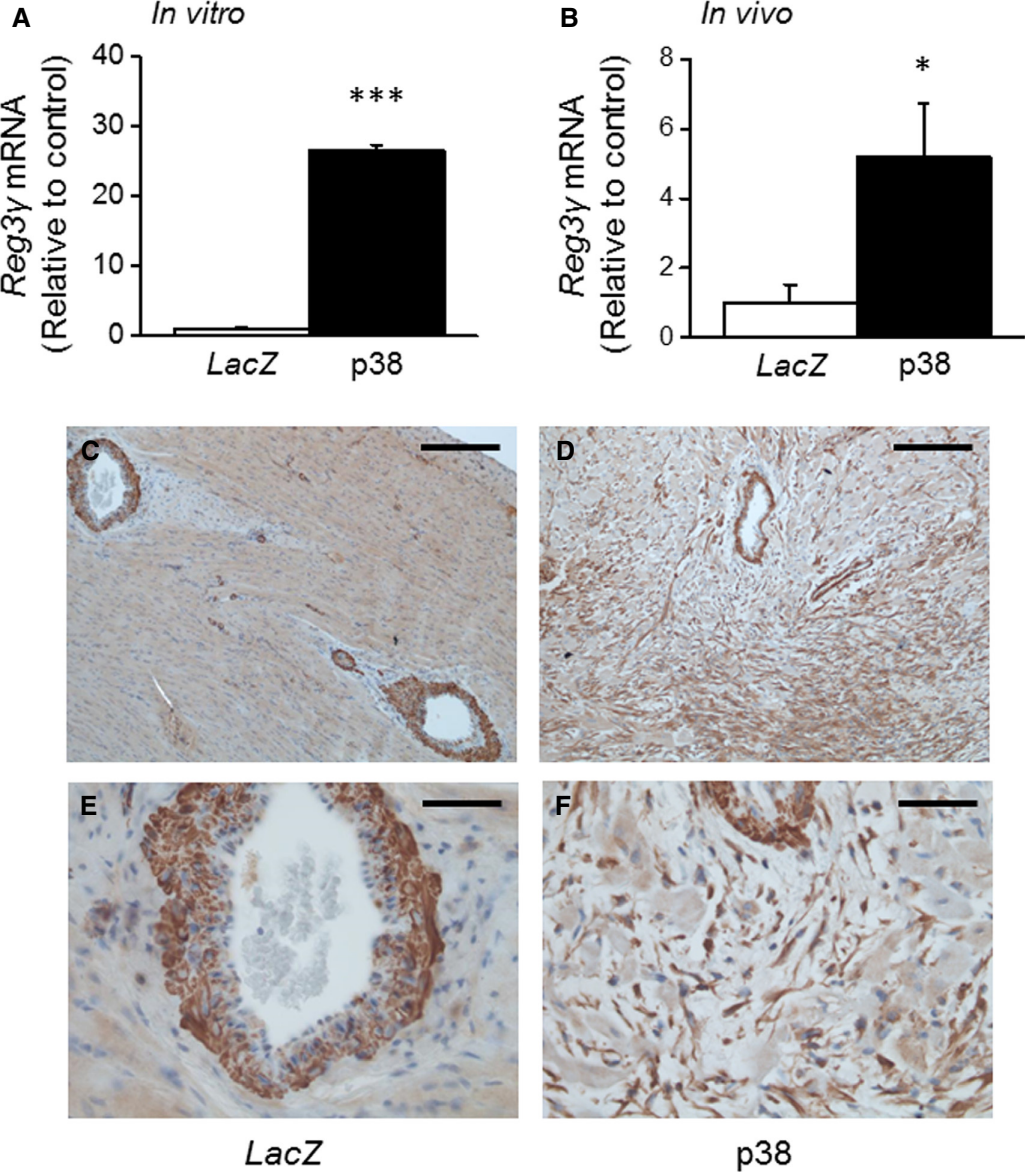
The effect of p38 MAPK overexpression on cardiac regenerating islet‐derived 3*γ* (Reg3*γ*) mRNA levels in vitro and in vivo. (A) Reg3*γ* mRNA levels from cultured NRCM transduced with recombinant adenoviruses overexpressing Mkk3bE and p38*α* or control virus *LacZ* at the virus amounts of 4 MOI (2 + 2 MOI in combination). (B) Left ventricular Reg3*γ* mRNA levels 3 days after Mkk3bE and p38*α* gene transfer into left ventricle in rats compared to *LacZ*‐injected hearts. White columns, control; black columns, p38*α* MAPK + Mkk3bE. Gene expression results measured by quantitative RT‐PCR are expressed as a ratio of Reg3*γ* mRNA to 18S mRNA, mean ± SEM (*n* = 10–18). *P < 0.05, ***P < 0.001 p38*α* + Mkk3bE versus control (Student's *t*‐test). The immunohistochemical staining of Reg3*γ* in the p38*α* + Mkk3bE‐treated hearts. (C, E) LacZ indicates LacZ‐injected group and (D, F) p38MAPK group treated with p38*α* and Mkk3bE viruses. The time point was 3 days. Magnification 10× (A, C), 40× (B, D). Scale bar 200 nmol L^−1^ (A, B), 50 nmol L^−1^ (C, D).

Finally, NRCMs were transduced with recombinant adenoviruses encoding either combination of MKK3bE and WTp38*α* or LacZ as a control virus. The overexpression of p38 MAPK caused 27.0‐fold increase (*P* < 0.001) in Reg3*γ* mRNA level (Fig. 5A). To determine whether p38 MAPK regulates cardiac Reg3*γ* expression also in vivo, p38 MAPK was overexpressed using adenovirus‐mediated combined MKK3bE and WTp38*α* gene delivery into left ventricular free wall of adult SD‐rats. The p38 MAPK overexpression increased Reg3*γ* mRNA (5.2‐fold; *P* < 0.05) levels at day 3 following gene transfer (Fig. 5B). To identify the cell types expressing Reg3*γ* in p38 MAPK overexpressing hearts, the immunohistochemical staining with Reg3*γ*‐antibody was performed. In the LacZ‐treated control hearts, Reg3*γ* was mainly expressed in the vascular smooth muscle cells, whereas a fairly weak signal was detected in inflammatory cells and proliferating fibroblasts (Fig. 5C–F). In MKK3bE plus WTp38*α*‐treated hearts enhanced Reg3*γ* immunostaining was observed in proliferating fibroblasts and myofibroblasts, in line with the localization of Reg3*γ* in Ang II‐treated hearts.

## Discussion

The secretory protein Reg3*γ* (also known as PAP III) is expressed in several tissues, and its expression is induced in various injuries and inflammatory conditions in gastrointestinal tract (Iovanna et al. 1992; Ogawa et al. 2003), skeletal muscle (Klasan et al. 2014) as well as in central and peripheral nervous system (Namikawa et al. 2005, 2006; Ampo et al. 2009; Kawahara et al. 2011; Klasan et al. 2014). However, almost nothing is known about cardiac Reg3*γ* gene expression, although increased Reg3*γ* expression has been repeatedly identified in cardiac gene expression profiling screens (Rysä et al. 2005; Tenhunen et al. 2006a; Watanabe et al. 2008; Liu et al. 2009). In this study, we are first to characterize the expression and regulation of Reg3*γ* in the heart. We found that Reg3*γ* gene expression is rapidly up‐regulated by post‐MI remodeling and Ang II in the adult rat heart in vivo. We showed that losartan treatment reduced left ventricular Reg3*γ* gene expression levels activated by Ang II. We also observed that multiple growth‐promoting factors and mechanical stretch directly activated Reg3*γ* mRNA levels in cultured cardiomyocytes. Finally, we established the role of p38 MAPK as an upstream regulator of cardiac Reg3*γ* gene expression.

This study indicates that Reg3*γ* is activated in the heart against acute stress. Reg3*γ* mRNA and protein levels were rapidly induced after MI produced by ligation of LAD in rats and in response to Ang II‐induced pressure overload. Compared to other Reg family members similar transient upregulation of Reg1 has been described in cardiomyocytes in response to acute MI both in rats and humans (Kiji et al. 2005), and in a pressure overload model generated by aortic constriction in rats (Kiji et al. 2005). Reg proteins have roles as anti‐inflammatory, antiapoptotic, and mitogenic agents (Parikh et al. 2012). Since Reg3*γ* gene expression was upregulated in myocardium post‐MI as well as in LPS‐induced cardiomyocytes in vitro, our findings suggest that Reg3*γ* is associated with inflammatory response in the heart.

Our study reveals that, in addition to myocardial injury, neurohumoral factors regulate Reg3*γ* transcription. We showed that hypertrophic and growth factors directly increased Reg3*γ* expression in neonatal cardiomyocytes suggesting that autocrine/paracrine factors as well as mechanical stretch are important regulators of Reg3*γ* in the heart. Mechanical stretch is coupled with the cellular release of ET‐1, which acts as a vasoactive substance in the development of fibrosis (Leask 2015) but also in cardiac overload‐induced hypertrophy (Yamazaki et al. 1999). In addition, mechanical stretch coupled cellular release of Ang II could mediate the increase in Reg3*γ* mRNA levels (Sadoshima et al. 1992; Liang and Gardner 1998). Mechanical stretch is also known to induce gene expression of other inflammatory mediators such as IL‐18, tumor necrosis factor‐like weak inducer of apoptosis (TWEAK) and its receptor Fn14 (Mustonen et al. 2010; Yoshida et al. 2014) as well as transcription factors involved in cardiac inflammatory response (Koivisto et al. 2014), but whether mechanical stress functions directly as a proinflammatory stimulus remains to be established. It is noteworthy that although the primary cultures derived from the adult rat heart would reflect better the situation in the adult heart, the limitation is that these cultured cells either lose their morphology or they lose their ability to undergo spontaneous contractions, if cultured under conditions where the rod‐shaped morphology is retained (Jacobson and Piper 1986; Mitcheson et al. 1998).

This study is first to show that p38 MAPK regulates cardiac Reg3*γ* expression. The p38 MAPK activation contributes significantly to inflammation and regulates production of several proinflammatory cytokines including IL‐6 and TNF*α* (Rose et al. 2010). We have previously shown that increased p38 MAPK activity causes massive cell proliferation and inflammation with development of fibrosis, diastolic dysfunction, and massive anterior wall thickening in the heart (Tenhunen et al. 2006a). Here we established the role of p38 MAPK as regulator of Reg3*γ* transcription in the heart both in vivo and in vitro using two experimental models. In cultured neonatal rat myocytes in vitro, pharmacological inhibition of p38 MAPK pathway caused a significant attenuation of both LPS and mechanical stretch‐induced upregulation of the Reg3*γ* gene expression. In addition, inhibition of p38 MAPK by SB2203580 reduced in part Ang II‐induced increase in Reg3*γ* mRNA levels in vivo. Furthermore, adenovirus‐mediated overexpression of p38 MAPK resulted in increased Reg3*γ* gene expression both in vivo and in vitro. In addition, in p38 MAPK overexpressing hearts showed increased Reg3*γ* immunoreactivity in proliferating fibroblasts and myofibroblasts. Previously it has been shown that STAT3, an important regulator of inflammatory response, regulates 3*β* expression in cardiomyocytes (Lörchner et al. 2015). Our results indicate that also other intracellular signaling molecules are involved in activation of Reg3 gene expression.

Our findings suggest that Reg3*γ* may also be involved in development of cardiac fibrosis along with the inflammatory response. Ang II infusion increased Reg3*γ* mRNA levels continuously during the 2 weeks’ follow‐up period, and in Ang II‐treated hearts Reg3*γ* immunoreactivity was mainly localized into the cardiac fibroblasts and myofibroblasts of the proliferating connective tissue. Furthermore, the localization of Reg3*γ* was similar to that of *α*‐SMA. In addition, Reg3*γ* gene expression was also induced in the hypertrophied hearts of old hypertensive rats that have established cardiac fibrosis (Boluyt and Bing 2000). Previously, Watanabe et al. (2008) have studied the subcellular mRNA expression pattern of Reg3*γ* and most of Reg3*γ* was detected in cardiomyocytes and noncardiomyocytic, noninflammatory (NCNI) cells (mainly fibroblasts, smooth muscle cells, and endothelial cells) compared to inflammatory cells. In this study, we were particularly interested in studying Reg3*γ* in cardiomyocytes. Mechanical stretch of both cardiac myocytes and cardiac fibroblasts has been shown to generate inflammatory mediators such as IL‐1*β* (Honsho et al. 2009), and when cardiomyocyte death occurs, myocardial damage is known to trigger chemokine and adhesion molecule production that attracts macrophages, monocytes, and neutrophils to the damaged area (Dobaczewski et al. 2010). Very recently, Lörchner et al. (2015) published an elegant study investigating the role of Reg3*β* in the heart, and identified Reg3*β* as an essential regulator of macrophage trafficking to the damaged heart. Inactivation of Reg3*β* impaired left ventricular function after infarction and resulted in increased matrix degradation due to insufficient neutrophil clearance by macrophages indicating that Reg proteins have a role in the control of macrophage trafficking during inflammatory responses and regenerative processes. Altogether, our data may imply that Reg3*γ* serves a cardioprotective role in the heart. However, further studies are needed to determine the functional role of Reg3*γ* in the heart.

## Physiological relevance

Our study shows that Reg3*γ* is associated with MI and pressure overload‐induced cardiac stress response and that Reg3*γ* is a p38 MAPK‐regulated gene. Altogether, our data suggest Reg3*γ* is associated with cardiac inflammatory signaling and offers a novel target of anti‐inflammatory therapeutic interventions.

## Conflict of Interest

None declared.
